# *tpo3* and *dur3*, *Aspergillus fumigatus* Plasma Membrane Regulators of Polyamines, Regulate Polyamine Homeostasis and Susceptibility to Itraconazole

**DOI:** 10.3389/fmicb.2020.563139

**Published:** 2020-12-16

**Authors:** Mingcong Chen, Guowei Zhong, Sha Wang, Jun Zhu, Lei Tang, Lei Li

**Affiliations:** ^1^Center for Global Health, School of Public Health, Nanjing Medical University, Nanjing, China; ^2^Key Laboratory of Vector Biology and Pathogen Control of Zhejiang Province, Huzhou Central Hospital, Huzhou University, Huzhou, China

**Keywords:** *Aspergillus fumigatus*, polyamine homeostasis, polyamine, reactive oxygen species, itraconazole susceptibility

## Abstract

*Aspergillus fumigatus* is a well-known opportunistic pathogen that causes invasive aspergillosis (IA) infections, which have high mortality rates in immunosuppressed individuals. Long-term antifungal drug azole use in clinical treatment and agriculture results in loss of efficacy or drug resistance. Drug resistance is related to cellular metabolites and the corresponding gene transcription. In this study, through untargeted metabolomics and transcriptomics under itraconazole (ITC) treatment, we identified two plasma membrane-localized polyamine regulators *tpo3* and *dur3*, which were important for polyamine homeostasis and susceptibility to ITC in *A. fumigatus*. In the absence of *tpo3* and/or *dur3*, the levels of cytoplasmic polyamines had a moderate increase, which enhanced the tolerance of *A. fumigatus* to ITC. In comparison, overexpression of *tpo3* or *dur3* induced a drastic increase in polyamines, which increased the sensitivity of *A. fumigatus* to ITC. Further analysis revealed that polyamines concentration-dependently affected the susceptibility of *A. fumigatus* to ITC by scavenging reactive oxygen species (ROS) at a moderate concentration and promoting the production of ROS at a high concentration rather than regulating drug transport. Moreover, inhibition of polyamine biosynthesis reduced the intracellular polyamine content, resulted in accumulation of ROS and enhanced the antifungal activity of ITC. Interestingly, *A. fumigatus* produces much lower levels of ROS under voriconazole (VOC) treatment than under ITC-treatment. Accordingly, our study established the link among the polyamine regulators *tpo3* and *dur3*, polyamine homeostasis, ROS content, and ITC susceptibility in *A. fumigatus*.

## Introduction

*Aspergillus fumigatus*, an opportunistic human pathogen in immunocompromised individuals, is a major invasive fungus. *A. fumigatus* spores are ubiquitous in the atmosphere. Inhalation exposure to these spores is associated with sinusitis, severe asthma, and even potentially lethal invasive infections for people with weakened immune systems, which are a challenge in global public health (Romani, 2011; van de Veerdonk et al., 2017; Latge and Chamilos, 2019). To date, triazoles are the first line of antifungal drugs for treating invasive aspergillosis (IA) (Marr et al., 2004; Garcia-Rubio et al., 2017; Espinel-Ingroff et al., 2019). Members of the largest family of antifungal triazole agents primarily include fluconazole (FLC), itraconazole (ITC), voriconazole (VOC), and posaconazole (POC). As one of the most commonly used systemic antifungal drugs, ITC has a free azole nitrogen, which competes with the heme moiety of cytochrome P450 for free oxygen, inhibiting the synthesis of ergosterol by 14-α-lanosterol demethylase (Cyp51/Erg11) in the fungal cell membrane, and side effects are infrequent (Rybak et al., 2019). With the widespread and continued use of azole drugs in the clinical and agriculture settings, the rate of *A. fumigatus* drug-resistant strains has steadily risen over the years (Snelders et al., 2008; Arendrup, 2014; Rybak et al., 2019). The development of azole drug tolerance is continuous and often makes antifungal treatment ineffective (Zavrel and White, 2015). Moreover, the number of IA-specific antifungal drugs is very limited; therefore their loss may cause challenges in patient management (Talbot et al., 2018). Consequently, a comprehensive understanding of the molecular mechanisms underlying azole resistance can provide theoretical support for the clinical therapy of IA.

Based on previous studies, several azole resistance mechanisms have been progressively disclosed in recent years, including alteration of the drug target *cyp51* encoding 14-α-lanosterol demethylase, activation of multidrug resistance (MDR) pumps, adaptation of cellular responses to drug-induced stress, and formation of biofilms (Cowen et al., 2014; Hagiwara et al., 2016; Rybak et al., 2019). Analysis of *A. fumigatus* clinical isolates and laboratory-derived mutants showed that the majority of resistance cases are related to *cyp51A* polymorphisms (Garcia-Rubio et al., 2018). Tandem insertions at the promoter of the *cyp51A* gene and/or some point mutations (e.g., TR34/L98H) in its coding region caused overexpression of Cyp51A and reduced its binding affinity to azoles, which could help *A. fumigatus* overcome azole activity and develop resistance (Garcia-Rubio et al., 2018; Rybak et al., 2019).

The overexpression or activation of MDR pumps is another very important mechanism responsible for azole resistance. Activated drug efflux systems pump out intracellular drugs, leading to a decrease in the concentration of drugs (da Silva Ferreira et al., 2004; Cowen et al., 2014; Ben-Ami et al., 2016; Meneau et al., 2016). The efflux system mainly consists of the ATP-binding cassette (ABC) and major facilitator superfamily (MFS), some of which have great clinical significance in the development of azole resistance in pathogenic fungi (Cowen et al., 2014). Drug efflux genes related to azole resistance have been extensively studied in *Candida* species (Zavrel and White, 2015). It is believed that there are at least 49 ABC and 278 MFS family genes encoding efflux transporters in the filamentous fungus *A. fumigatus* (Rajendran et al., 2011). Whether in susceptible strains exposed to azoles or in azole-resistant isolates, many transporters elevate their expression levels (Fraczek et al., 2013; Long et al., 2018). However, upregulation of transporters does not always confer resistance to the susceptible strains, possibly reflecting secondary effects of azoles on metabolism (Fraczek et al., 2013). Therefore, the relationship between the induction of transporters by drugs and drug resistance needs to be further confirmed by manipulation, such as overexpression or gene knockout. Reportedly, overexpression of the ABC transporters *abcA*, *abcB*/*cdr1B*, *AfuMDR4*, *atrF*, and the MFS transporter *AfuMDR3* resulted in azole resistance (Nascimento et al., 2003; Fraczek et al., 2013; Meneau et al., 2016; Paul et al., 2017).

Recently, an association between cellular metabolites and azole resistance has been found in several studies; for instance, melanin, sphingolipids, extracellular matrix (ECM), and polyamines have been proven to be related to azole resistance in fungi (van de Sande et al., 2007; Fanning and Mitchell, 2012; Liao et al., 2015; Long et al., 2018). Polyamines are small aliphatic hydrocarbon molecules containing two or more amine groups, including putrescine (Put), spermidine (Spd), and spermine (Spm). Polyamines play a pivotal role in growth and stress tolerance in a range of organisms (Michael, 2016). In *Cryptococcus neoformans*, defects of the polyamine synthesis impaired capsule formation, melanin production, growth rate, and virulence (Kingsbury et al., 2004). In *Candida albicans* and *Candida glabrata*, polyamine biosynthesis and transport influenced the resistance to several specific antifungal agents by different mechanisms (Tati et al., 2013; Costa et al., 2014; Liao et al., 2015). Transregulators of polyamine uptake regulated the activities of cationic antifungal peptides in *Saccharomyces cerevisiae* and *Fusarium oxysporum* (Bleackley et al., 2014). In *A. fumigatus*, polyamines played a role in iron homeostasis and cell wall integrity, which is vital for fungal survival when exposed to stress conditions (Jain et al., 2011; Beckmann et al., 2013; Schafferer et al., 2015). However, the relationship between cellular polyamine homeostasis and azole resistance in filamentous fungi has yet to be elucidated.

With the aim of identifying cellular metabolites and their related regulatory genes involved in the responses to azole-induced stress in *A. fumigatus*, through untargeted metabolomics and transcriptomics analysis, we identified intracellular accumulated spermidine and two putative polyamine transporter genes, *tpo3* (AFUB_101650/AFUA_4G01140) and *dur3* (AFUB_005210/AFUA_1G04870). Using reverse genetics, we further demonstrated that *A. fumigatus* plasma membrane-localized *tpo3* and *dur3* played important roles in susceptibility to ITC via a potential mechanism (*tpo3* and *dur3* → polyamine homeostasis → ROS content → ITC susceptibility). More importantly, our study will provide new insights into the function of polyamine homeostasis and ROS content in *A. fumigatus* adaptation to the azole stress.

## Materials and Methods

### Strains, Media, and Culture Condition

All the *A. fumigatus* strains used in this study are shown in Supplementary Table S1. The media used in this study included YAG (0.5% yeast extract, 2% glucose, 0.1% 1,000 × trace elements, 2% agar), YUU (YAG supplemented with 5 mM uridine and 10 mM uracil), and minimal medium (MM) (1% glucose, 0.1% 1,000 × trace elements, 5% 20 × salts [pH 6.5]) with or without 5 mM uridine and 10 mM uracil (MMUU). For the antifungal test, conidia were harvested, adjusted to 1 × 10^7^ spores ml^–1^ in sterile water, and then inoculated on indicated medium in the absence or presence of different concentrations of ITC, VOC, caspofungin (CS), polyamines, or polyamine inhibitor. ITC and VOC were prepared in dimethyl sulfoxide. CS, polyamines, and α-difluoromethylornithine (DMFO)/eflornithine were prepared in sterile deionized water.

### Metabolomics

LC-MS analysis was performed as described previously (Long et al., 2018). In brief, spores of the A1160^C^ strain were divided into WT (A1160^C^) and WT-1ITC (A1160^C^ plus 1 μg ml^–1^ ITC) two groups (each group contained six biological replicates). For each biological replicate, 3 × 10^7^ spores were cultured in 300 ml liquid YAG media at 220 rpm and 37°C for 35 h. Then, the samples were continued to incubate in the presence or absence of 1 μg ml^–1^ ITC for 1 h. Mycelia were harvested and lyophilized, and 100 mg accurately weighed sample was transferred to a 1.5 ml Eppendorf tube. Then, 2 ml of methanol-water [4:1 (V/V)] and 400 μl of chloroform were added to each sample, and 20 μl of 2-chloro-L-phenylalanine (0.3 mg ml^–1^) dissolved in methanol as an internal standard. An ultrasonic homogenizer was used to break up the cells for 6 min at 500 W. All of the mixtures of each sample were then extracted by ultrasonication for 20 min. The extracts were centrifuged at 13,000 × *g* at 4°C for 10 min. Quality control samples were prepared by mixing aliquots of all samples to serve as a pooled sample. Then, 500 μl portions of the supernatants of each tube were sampling in a 1290 Infinity UHPLC system coupled with a 6538 UHD QTOF mass spectrometer (LC-MS) (Agilent Technologies, Inc., Los Angeles, CA). Differential metabolites were identified using the Human Metabolome Database (HMDB) and the METLIN metabolite database. The differential metabolites were selected on the basis of the combination of a statistically significant threshold of variable influence on projection (VIP) values obtained from the OPLS- DA model and *p*-values from a two-tailed Student’s *t*-test on the normalized peak areas, where metabolites with VIP values larger than 1.0 and *p*-values less than 0.05 were included, respectively. More details about metabolomics and the raw data were deposited in the EMBL-EBI MetaboLights database^1^ with the identifier MTBLS1739 and the url https://www.ebi.ac.uk/metabolights/MTBLS1739.

### Deletion and Complementation of *tpo3* and/or *dur3*

All primers used in this study are displayed in Supplementary Table S2. For deletion of *tpo3* gene, fusion PCR approach was employed to generate the fragment sequentially containing upstream fragment, *Neurospora crassa Ncpyr4* selectable marker, and downstream fragment (Jiang et al., 2014). Upstream flanking sequences about 884 bp that corresponded to the region immediately upstream of the *tpo3* start codon, were amplified from the A1160 genomic DNA (gDNA) using the primers *tpo3* P1 + *tpo3* P3. Downstream flanking sequences about 960 bp that corresponded to the regions immediately downstream of the *tpo3* stop codon, were amplified with the primers *tpo3* P4 + *tpo3* P6. The *N. crassa Ncpyr4* was used as a selectable nutritional marker for fungal transformation and amplified from plasmid pAL5 (Jiang et al., 2014) with the primers pyr4 F + pyr4 R. These three fragments were then mixed as templates and used in a fusion PCR with primers *tpo3* P2 + *tpo3* P5 to construct the *tpo3* deletion cassette. Then, the *tpo3* deletion cassette was transformed into the wild type A1160 to achieve homologous recombination. A diagnostic PCR assay was performed to identify the deletion of the *tpo3* gene with the primers *tpo3* P2 + Diag*tpo3*. Deletion of *dur3* was generated and identified by the same method in the A1160 background using *Ncpyr4* as a selectable nutritional marker. For double deletions of *tpo3* and *dur3*, a similar strategy was used. The *dur3* deletion cassette was generated using upstream fragment, the hygromycin B resistance gene *hph* selectable marker amplified from the plasmid pAN7-1 (Cai et al., 2016) using primers hph F + hph R, and downstream fragment. The *dur3* deletion cassette was then transformed into the background of an *tpo3*-deleted mutant and transforments were screened using 300 μg ml^–1^ of hygromycin B. All the deletion strains were confirmed by diagnostic PCR. For the complementation of *tpo3* and *dur3* null mutant, the full-length *tpo3* and *dur3* genes were amplified using the primer pairs tpo3-com S1/S2 and dur3-com S1/S2, respectively, which contain native promoters, the 5′UTRs, gene coding sequences, and the 3′UTRs. The two complementation cassettes were subsequently transformed into *tpo3*- and *dur3*-null mutants, respectively, and transforments were screened using 5-fluoroorotic acid as previously described (Jiang et al., 2014).

### Overexpression of *tpo3* and *dur3*

We used the following strategy to overexpress *tpo3* and *dur3*. Briefly, using the primer pairs OE-tpo3 F + OE-tpo3 R and OE-dur3 F + OE-dur3 R, the Open Reading Frames (ORFs) of *tpo3* and *dur3* were amplified from the A1160 gDNA. Purified fragments were then subcloned into the *Cla*I site of pBARGPE (Song et al., 2016) to generate the overexpression plasmids. Then, the plasmids were transformed into A1160 strain.

### Construction of Tpo3 and Dur3 GFP-Tagged Strains

To label Tpo3 with a green fluorescence protein (GFP) tag at the C terminus, we first amplified the *tpo3* 3′ flanking sequence (without the termination codon) and *tpo3* downstream flanking sequence from A1160 strain gDNA using the primers tpo3-gfp P1 + tpo3-gfp P3 and tpo3-gfp P4 + tpo3-gfp P6, respectively. Then, a DNA fragment containing a Gly-Ala linker, GFP coding sequence, and *A. fumigatus AfpyrG* was amplified from the plasmid pFNO3 (Song et al., 2016) using the primers gfp + pyrg F and gfp + pyrg R. The three DNA fragments were used as a template to generate a final construct via fusion PCR using the primers tpo3-gfp P2 + tpo3-gfp P5. The PCR product was then transformed into the A1160 strain. A similar approach was used to generate strain expressing Dur3-GFP at the C terminus.

### Intracellular Polyamine Accumulation Assessment

Intracellular polyamine assessment was performed as a previous study (Costa et al., 2014). Spores (1 × 10^7^) of indicated strains were cultured in 100 ml of liquid MM, MMUU, or YAG media supplemented with or without 10 mM DFMO at 37°C and 220 rpm for 20 h. Then, mycelia were filtered and collected. To extract polyamines, 100 mg mycelia were resuspended in 1 ml of 10% trichloroacetic acid, placed in an ice bath and broken with an ultrasonic crusher. After centrifugation, the supernatants were transferred into another 10 ml centrifuge tube and polyamines were derivatized by the addition of 1 ml of 2 M NaOH and 10 μl of benzoyl chloride for 30 min at 37.5°C, and oscillated every 5 min. The benzoylation was stopped by adding 2 g NaCl, and the derivatized polyamines were extracted with 1 ml ethyl ether, blown dry by nitrogen and solubilized in 1 ml methanol. Derivatized polyamine extracts were then collected and analyzed by HPLC on a 160 mm × 4.6 mm C18 column (C/N 5020-07345 S/N 6JR97089 end capped 5 mm) at 42°C. Using 50% (v/v) methanol in water for 0.5 min, followed by a linear gradient methanol from 50% (v/v) to 85% (v/v) in water for 6.5 min at a flowrate of 0.6 ml min^–1^. This was followed by an isocratic elution at 85% (v/v) methanol in water for 5 min at a flowrate of 0.6 ml min^–1^. Finally, a decrease over 2 min to 50% (v/v) methanol in water at 0.6 ml min^–1^. Peaks corresponding to polyamines were detected using absorption spectrophotometry at a wavelength of 254 nm. The results indicate that the respective retention times for Spm, Spd, and Put were 13.6, 12.8, and 11.0 min. The standard curve was prepared with 20, 40, 100, 200, 400, and 500 μM of Spm, Spd or Put. The detectable concentration range of Spm, Spd, and Put in samples were 40∼200, 100∼500, and 20∼100 μM, respectively. The final concentration units of Spm, Spd, and Put in samples were converted to μM g^–1^ (Molar mass per Hypha weight).

### Cellular Drug Detection

Cellular drug was measured as described previously (Long et al., 2016). Briefly, 1 × 10^7^ spores of the indicated strains were cultured in 100 ml of liquid MMUU medium at 37°C and shaken at 220 rpm for 20 h. Then, a final concentration of 1 μg ml^–1^ ITC was added to the media and incubated for 1 h. Mycelia were harvested and washed with distilled water to remove the extracellular ITC, and then lyophilized. Approximately 100 mg of lyophilized mycelia was incubated in 1 ml of 50% (v/v) methanol in water and homogenized using ceramic beads. The cell debris and ceramic beads were then removed by centrifugation at 13,000 × *g* for 10 min. The supernatant was analyzed using HPLC on an AQ-C18 column (250 mm by 4.6 mm, 5 μm) with an isocratic profile at 65% (v/v) acetonitrile in phosphate buffer at a flow rate of 1 ml min^–1^. Peak corresponding to ITC was detected using absorption spectrophotometry at a wavelength of 265 nm. The results indicate that the retention times for ITC was 8.10 min. The standard curve was prepared with 1, 2, 4, 8, 16, and 32 μg ml^–1^ of ITC. The detectable concentration range of ITC in samples is 8∼16 μg ml^–1^. The final concentration unit of ITC in the samples is converted to μg g^–1^ (Drug weight per Hypha weight).

### Real-Time PCR (qRT-PCR)

For qRT-PCR analysis, 1 × 10^7^ spores of *A. fumigatus* were inoculated into 100 ml of liquid MMUU media and shaken on a rotary shaker at 220 rpm at 37°C for 20 h. Total RNA of the indicated strains was isolated from fresh mycelia by using TRIzol as described in the manufacturer’s instructions. The digestion of genomic DNA and the synthesis of cDNA were performed using HiScript R II Q RT SuperMix for qPCR kit (Vazyme) as its instruction. qRT-PCR was executed by ABI One-step fast thermocycler (Applied Biosystems) with SYBR Premix Ex TaqTM (TaKaRa). Relative expression levels were calculated using the 2^–Δ^ ^Δ^ ^Ct^ method (Livak and Schmittgen, 2001).

### Library Construction and RNA Sequencing (RNA-Seq) Procedures

For RNA-seq analysis, spores of the A1160^*C*^ strain were divided into WT (A1160^C^) and WT-1ITC (A1160^C^ plus 1 μg ml^–1^ ITC) two groups (each group contains three biological replicates) and 1 × 10^7^ spores were inoculated into 100 ml liquid YAG media and shaken on a rotary shaker at 220 rpm at 37°C for 16 h, with a subsequent 1-h regrowth in the same condition or a subsequent 1-h shift into 1 μg ml^–1^ ITC condition. These two groups of RNA samples were prepared to perform transcriptome analysis by the RNA-seq approach. Total RNA was isolated from samples using RNeasy mini kit (Qiagen, Germany). Paired-end libraries were synthesized by using the TruSeq^®^ RNA Sample Preparation Kit (Illumina, United States) following TruSeq^®^ RNA Sample Preparation Guide. Briefly, the poly-A containing mRNA molecules were purified using poly-T oligo-attached magnetic beads. Purified libraries were quantified by Qubit^®^ 2.0 Fluorometer (Life Technologies, United States) and validated by Agilent 2100 bioanalyzer (Agilent Technologies, United States) to confirm the insert size and calculate the mole concentration. Cluster was generated by cBot with the library diluted to 10 pM and then were sequenced on the Illumina HiSeq X-ten (Illumina, United States). The library construction and sequencing was performed at Shanghai Biotechnology Corporation.

### Transcriptome Analysis

The raw Illumina sequencing data were deposited in SRA^2^ at NCBI with accession numbers SRX8354093 to SRX8354098. Sequencing raw reads were preprocessed by filtering out rRNA reads, sequencing adapters, short-fragment reads < 25bp and Q20 < 20% (percentage of sequences with sequencing error rates <1%). We used Hisat2 (version: 2.0.4) to map the cleaned reads to the *A. fumigatus* A1163 reference genome. After genome mapping, Stringtie (version: 1.3.0) was run with a reference annotation to generate FPKM values for known gene models. Differentially expressed genes were identified using edgeR. The *p*-value significance threshold in multiple tests was set by the false discovery rate (FDR). The fold-changes were also estimated according to the FPKM in each sample. The differentially expressed genes were selected using the following filter criteria: FDR ≤ 0.05 and fold-change ≥ 2.

### Intracellular ROS Detection and Microscopic Observations

For ROS microscopic observations, a previous study was used (Satish et al., 2019). Spores (1 × 10^5^) of the indicated strains were cultured in 2 ml of liquid YAG medium in a confocal dish supplemented with or without 10 mM DFMO or 0.3 mM Spd at 37°C for 10 h. After the culture was completed, hyphae were washed by PBS for 3 times. DCFH-DA (2, 7-Dichlorofuorescin Diacetate) was added at a final concentration of 15 μm, and then incubated at 37°C for 30 min without light. Then, hyphae were washed by PBS for 3 times. 200 μl of 1 μg ml^–1^ ITC in PBS was added to the dish, followed by incubation at 37°C for 3 h. Images were observed and photographed by Axio Imager A1 microscope (Zeiss, Jena, Germany). For intracellular ROS quantitation, 200 μl suspension of 1 × 10^5^ conidia ml^–1^ in YAG medium supplemented with or without 10 mM DFMO or 0.3 mM Spd was incubated in black, clear bottom 96-well Costar plate at 37°C for 10 h. After staining and ITC treatment as above used, the fluorescence intensity was measured with an excitation filter at 495 nm and an emission filter at 530 nm in a microtiter plate reader (Infinite M200 Pro; Tecan, Switzerland) at 37°C. Unstained cells were used as a blank.

### Antifungal Susceptibility Testing

The conidia of tested *Aspergillus* strains were harvested, adjusted to 1 × 10^7^ spores per ml in phosphate buffer saline, and then inoculated into liquid RPMI 1640 media plus 5 mM uridine and 10 mM uracil in the presence of different concentrations of the azole antifungals ITC. ITC were prepared in the stock solution with dimethyl sulfoxide. Broth microdilution was performed according to CLSI-M38-A2. Briefly, 2-fold serial drug dilutions were prepared in flat-bottom 96-well microtiter plates (100 μl per well), Drug-free wells were used as controls. Each well was inoculated with 100 μl of freshly isolated spores (1 × 10^4^ Conidia per ml) suspended in RPMI 1640 plus 5 mM uridine and 10 mM uracil. After 48 h of incubation at 35°C, the MIC was recorded as the lowest drug concentration at which no growth was observed.

## Results

### *A. fumigatus* Accumulates Spermidine After Stimulation With ITC

**FIGURE 1:**
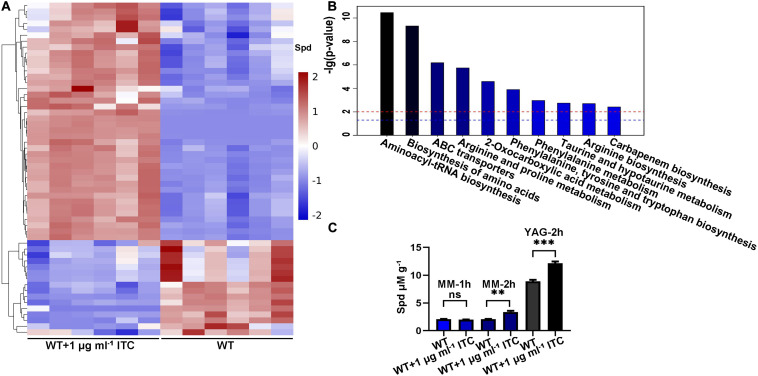
ITC treatment increases the intracellular Spd accumulation. **(A)** Hierarchical clustering dendrogram of differential metabolites including Spd between groups of WT (A1160^C^) and WT-1ITC (A1160^C^ plus 1 μg ml^– 1^ ITC). Red, upregulation; blue, downregulation. **(B)** Histogram of the different pathways enriched between the two groups according to the -lg (*p*-value) values and KEGG pathway analysis. The blue and red horizontal line indicates *p* = 0.05 and *p* = 0.01, respectively. **(C)** Intracellular Spd concentrations in WT and WT plus 1 μg ml^– 1^ ITC samples cultured in the indicated media and treated by ITC at different time points. Statistical analyses were performed by one-way ANOVA with unpaired Student’s *t*-test and the data were presented as the means ± SD of three biological samples (**p < 0.01; ***p < 0.001).

To analyze and identify the differentially altered cellular metabolites that can play important roles in the adaptation of *A. fumigatus* to ITC exposure, we performed a comparative metabolomics study. Two groups of samples were collected. In group 1, conidia from the wild-type strain (A1160^C^) were cultured in YAG liquid media for 35 h and then exposed to 1 μg ml^–1^ ITC for an additional 1 h before metabolite extraction (WT-1ITC). In group 2, conidia from the wild-type strain (A1160^C^) were cultured in YAG liquid media for 36 h and served as the control (WT). Then, we adopted ultrahigh-performance liquid chromatography coupled with quadrupole time-of-flight MS (UHPLC/Q-TOF MS) for metabolomics profiling. After principal component analysis (PCA), partial least-squares discriminant analysis (PLSDA) (Supplementary Figure S1), and hierarchical clustering analysis (Figure 1A), the differently changed intracellular metabolites between the two groups of samples were identified and evaluated. Overall, approximately 56 metabolites (variable importance in projection [VIP] > 1; *p* < 0.05) were reported. Furthermore, 40 and 16 small molecules were up- and downregulated by >1.17- and <0.87-fold, respectively, in the treated strains compared with the untreated strains (Supplementary Table S3). Kyoto Encyclopedia of Genes and Genome (KEGG) pathway^3^ analysis predominantly mapped the representative differentially abundant metabolites onto 10 principal pathways (Figure 1B). Apparently, amino acid-related metabolism (aminoacyl-tRNA biosynthesis, biosynthesis of amino acids, arginine, and proline metabolism) and ABC transporters (spermidine and amino acid transporters) were the two largest pathways upregulated in the ITC-treated group compared with the untreated group. In addition, the levels of some interesting metabolites, e.g., LysoPC (16:0), LysoPC [18:3(6Z, 9Z, 12Z)], LysoPC [20:4(5Z, 8Z, 11Z, 14Z)], LysoPC [18:1(11Z)], LysoPC [16:1(9Z)], and PC [16:0/20:5(5E, 8E, 11E, 14E, 17E)], were significantly decreased, implying a possible correlation with adaptation to ITC.

Intriguingly, the level of spermidine showed a 1.35-fold increase under ITC stimulation (Figure 1A). Spermidine (Spd), putrescine (Put), and spermine (Spm) are collectively known as polyamines. The biosynthetic pathway of polyamines starts with arginine and first generates ornithine, followed by the formation of Put and Spd, and eventually yields Spm (Walters et al., 1997). Given the importance of polyamines in the microbial drug-resistance process, a verification experiment was performed by high-performance liquid chromatography (HPLC) using Spd as the standard. The Spd content was 1.5- and 1.3-fold higher than that of the normal control after induction by 1 μg ml^–1^ ITC in MM and YAG media, respectively (Figure 1C). Based on the fact that ITC stimulation is always accompanied by an increase in Spd, we assumed that the elevation of Spd levels is a potentially important mechanism for *A. fumigatus* generating azole tolerance or resistance.

### Polyamines Are Involved in ITC Resistance and Antifungal Sensitivity

**FIGURE 2:**
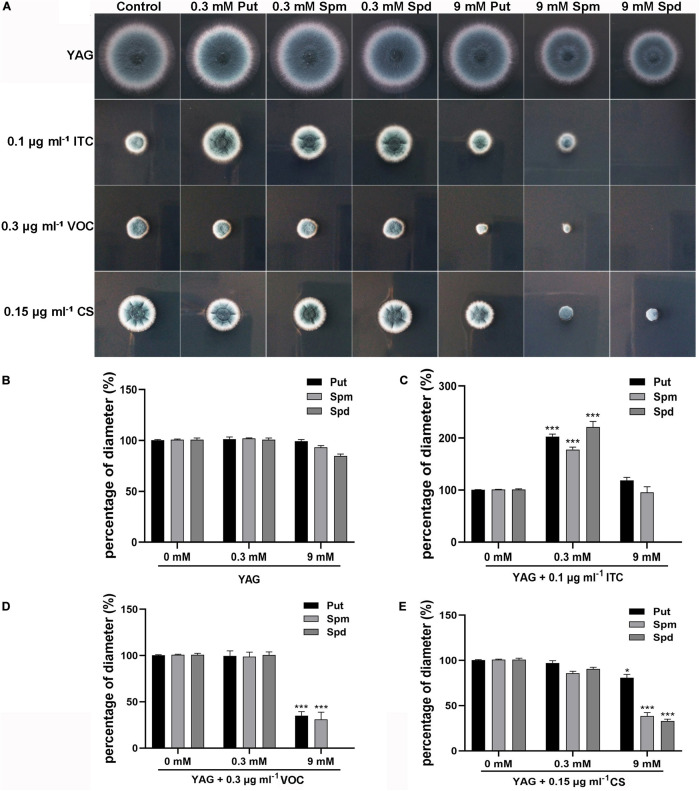
Extracellular addition of polyamines (pH 6.5–7.0) at the indicated concentrations affected resistance or sensitivity of the wild-type strain (A1160^C^) to the antifungal ITC, VOC, or CS. **(A)** Strains were tested on YAG plates and were cultured at 37°C for 2 days. The experiment was repeated three times. **(B)** The percentage of diameter of A1160^C^ cultured on YAG plates with concentrations of polyamines when compared to the diameter of A1160^C^ on YAG plates. The percentage of diameter of A1160^C^ cultured on YAG plates with concentrations of polyamines combined with 0.1 μg ml^– 1^ ITC **(C)**, 0.3 μg ml^– 1^ VOC **(D)**, or 0.15 μg ml^– 1^ CS **(E)** when compared to the diameter of A1160^C^ on YAG plates with 0.1 μg ml^– 1^ ITC, 0.3 μg ml^– 1^ VOC, or 0.15 μg ml^– 1^ CS, respectively. Statistical analyses were performed by one-way ANOVA with unpaired Student’s *t*-test and the data were presented as the means ± SD of three biological samples (*p < 0.05; ***p < 0.001).

Considering the finding that ITC treatment resulted in mild accumulation of Spd in mycelial cells, we sought to determine whether there is a relationship between polyamine utilization and antifungal drug resistance. To this end, the antifungal (ITC, VOC, and caspofungin [CS]) susceptibility of the A1160^C^ strain was tested in media supplemented with different concentrations of exogenous polyamines (Put, Spm, and Spd). As shown in Figure 2A, we divided the colony phenotypes into two classes according to the contribution of 0.3 mM polyamines to azole tolerance; this treatment, had no detectable effect on colony growth of the A1160^C^ strain when administered alone at this concentration. In class I, 0.3 mM polyamines can help *A. fumigatus* acquire ITC resistance (Figure 2C), indicating that the combination of polyamine additives with ITC exhibits an antagonistic effect. However, when 0.3 mM polyamines were combined with VOC or CS, no detectable effect on colony growth was observed in class II (Figures 2D,E), suggesting that there was no obvious interrelationship between 0.3 mM polyamines and these two antifungal agents. To our surprise, an adverse effect was observed in the assay with the addition of high concentrations of Spm (9 mM) and Spd (9 mM), which caused a slight defect in colony growth when used alone (no significance was found as shown in Figure 2B). Furthermore, the growth of *A. fumigatus* was almost inhibited by the presence of ITC, VOC, or CS and high-dose Spd (9 mM) compared to that of antifungal agent treatment alone (Figure 2). In this way, a synergetic effect against *A. fumigatus* was achieved. Therefore, an exogenous moderate dose of polyamines can confer ITC resistance on *A. fumigatus*, while an overdose of polyamines acts in synergy with antifungal drugs to confer antifungal activity against *A. fumigatus*.

### Combination of DFMO/Eflornithine and ITC Enhanced the Activity Against *A. fumigatus*

**FIGURE 3:**
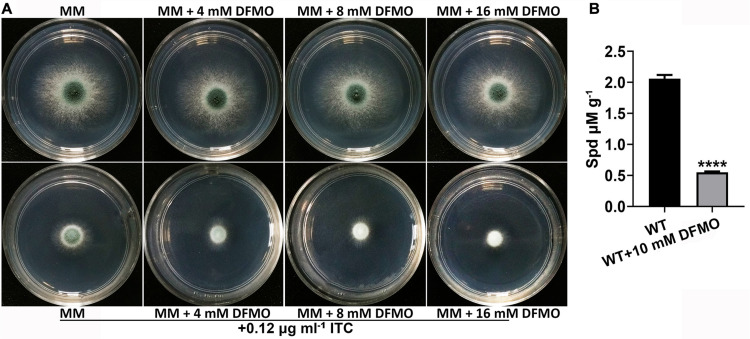
Combination of DFMO and ITC enhanced the activity against *A. fumigatus*. **(A)** Comparison of colonies of the ITC-untreated A1160^C^ strain (up panels) and ITC-treated A1160^C^ strain (down panels) on MM plates in the absence or presence of the indicated concentrations of DFMO at 37°C for 48 h. **(B)** Addition of DFMO, a polyamine biosynthesis inhibitor, at 10 mM reduced Spd production in A1160^C^. Statistical analyses were performed by one-way ANOVA with unpaired Student’s *t*-test and the data were presented as the means ± SD of three biological samples (****p < 0.0001).

It has been reported that the combination of polyamine biosynthesis inhibitors and amphotericin B enhances the activity against *C. albicans* biofilms (Liao et al., 2015). To investigate whether a combination of polyamine biosynthesis inhibitors and ITC can enhance the inhibitory effect on the growth of *A. fumigatus*, we tested the susceptibility to ITC of the A1160^C^ strain when it was cultured on media supplemented with or without different concentrations of DFMO/eflornithine (Beckmann et al., 2013; Liao et al., 2015). As shown in Figure 3A, inhibition of polyamine biosynthesis in *A. fumigatus* caused sensitivity to ITC. HPLC analysis confirmed the reduced intracellular polyamine content under this condition (Figure 3B). Collectively, these results suggested that inhibition of polyamine biosynthesis reduced the level of intracellular polyamines and enhanced the activity of ITC against *A. fumigatus*.

### *A. fumigatus* Upregulates the Transcription of Putative Transporter Genes During Induction by ITC

**FIGURE 4:**
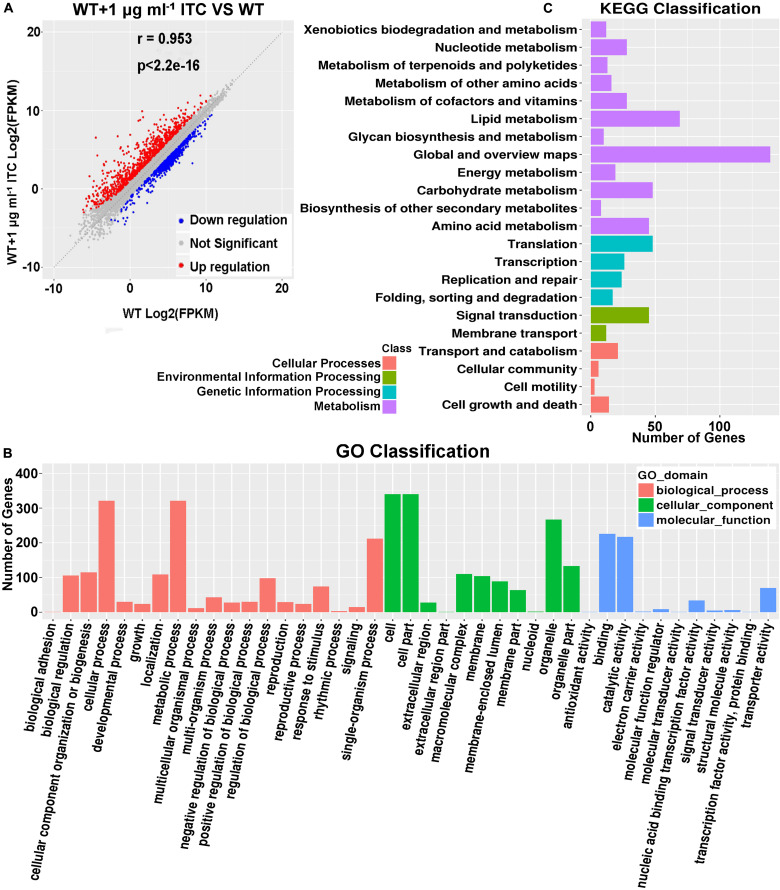
Identification of *tpo3* and *dur3* with changes at transcription levels after ITC treatment. **(A)** A scatter plot showing the differential gene expression between the ITC-treated and untreated groups. Red, upregulation; blue, downregulation; gray, not significant. **(B)** GO term enrichment analysis of RNA-seq Data. **(C)** Genes were enriched in different categories by KEGG analysis. The *x*-axis represents the number of enriched genes among the total genes in different categories.

Our metabolomics data showed that altered Spd and amino acid contents were mapped to ABC transporters, which led us to propose that ITC-induced stress probably increases the transcriptional levels of corresponding metabolic pathway-related genes. To this end, a transcriptome sequencing (RNA-seq)-based approach was used. Two groups of samples were prepared. In group 1, conidia from the wild-type strain (A1160^C^) were cultured in MM liquid media for 16 h and then exposed to 1 μg ml^–1^ ITC for an additional 1 h before RNA extraction (WT-1ITC). In group 2, conidia from the wild-type strain (A1160^C^) were cultured in MM liquid media for 17 h and served as the control (WT). Our comparative RNA-seq analysis revealed that the gene expression profile of the A1160^C^ strain was strongly affected by ITC stimulation. Among the total 9,593 *A. fumigatus* transcripts, 1,465 were significantly regulated (*P* ≤ 0.05, −1 ≥ log_2_FC ≥ 1) after ITC treatment (Supplementary Table S4). Of the 1,465 differentially expressed genes between the two experimental groups, 882 were upregulated and 583 were downregulated (Figure 4A and Supplementary Table S4). As shown by the Gene Ontology (GO) functional enrichment analysis, the differentially expressed genes were primarily involved in the binding, catalytic activity, transporter activity, nucleic acid binding transcription factor activity, etc. (Figure 4B). By using KEGG pathway classification, we found that the levels of components of lipid metabolism, carbohydrate metabolism, amino acid metabolism, signal transduction, and membrane transport (environmental information processing) were significantly changed in the ITC-treated cells compared to the untreated cells (Figure 4C).

Of interest, among the genes related to polyamine synthesis and transport, only two putative efflux transporters were significantly changed, AFUB_101650 (AFUA_4G01140 in the Af293 strain) and AFUB_005210 (AFUA_1G04870 in the Af293 strain). AFUB_101650 is a predicted MFS multidrug and Spm transporter that showed a 3.03-fold increase in transcriptional level. AFUB_005210 putatively plays roles in Put, Spd, and urea transport and had a 2.15-fold increase in expression in the ITC-treated cells (Supplementary Table S5). The orthologs AFUB_101650 and AFUB_005210 are named *TPO3* and *DUR3* in *S. cerevisiae*, respectively. Consequently, we named AFUB_101650 *tpo3* and designated AFUB_005210 *dur3* in *A. fumigatus* in this study. In summary, our RNA-seq results suggested that ITC treatment induced transporter activity, including the putative transporters *tpo3* and *dur3*, which might regulate polyamines or drug transmembrane transport.

### Deletion or Overexpression of Plasma Membrane-Located *tpo3* and *dur3* Altered the Susceptibility to ITC of *A. fumigatus*

**FIGURE 5:**
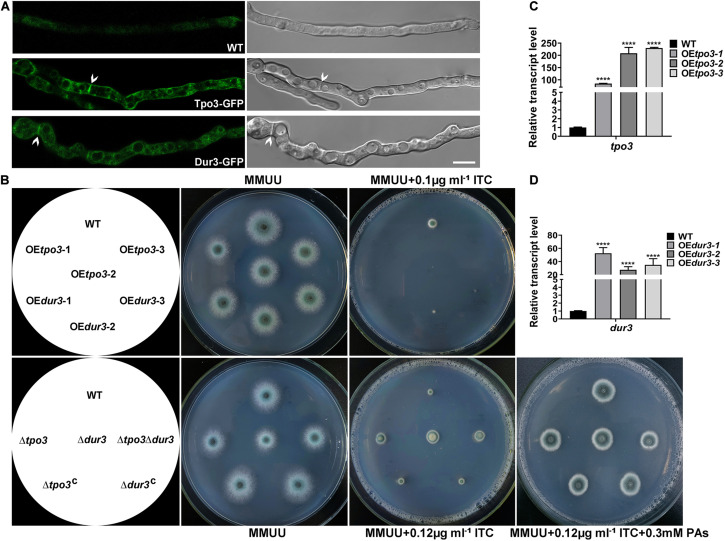
Cell membrane-located Tpo3 and Dur3 are responsible for susceptibility to ITC of *A. fumigatus*. **(A)** Confocal microscopy of vegetable hyphae expressing Tpo3-GFP or Dur3-GFP. The images are overlays of the green and DIC channels (right panels). Arrows indicate Tpo3-GFP and Dur3-GFP are assembled to septa. Bars, 10 μm. **(B)** Comparison of the susceptibility to ITC of the reference strain A1160, OE*tpo3*, OE*dur3*, Δ*tpo3*, Δ*dur3*, and Δ*tpo3Δdur3* mutants. Strains were grown on MMUU agar plates supplemented with or without ITC at the indicated concentrations and incubated at 37°C for 2 days. The transcript levels of *tpo3* **(C)** and *dur3* **(D)** in the *tpo3*- and *dur3*-overexpressing mutants and reference strain A1160 were detected by real-time PCR. Beta-tubulin gene *tubA* was used as endogenous reference gene, and gene expression is presented as the fold change relative to the wild-type strain. Statistical analyses were performed by one-way ANOVA with unpaired Student’s *t*-test and the data were presented as the means ± SD of three independent experiments (****p < 0.0001).

Using BLASTP and TMHMM analysis tools^4^ to predict the topology of amino acid sequences, we revealed that Tpo3 possesses a highly conserved MFS domain of its putative orthologs and consists of 12 transmembrane α-helical segments. Dur3 belongs to the SLC5-6-like_sbd superfamily, containing 15 transmembrane α-helical segments (Supplementary Figure S2). We hypothesized that Tpo3 and Dur3, as putative transporters or regulators, are located in the plasma membrane of *A. fumigatus* hyphal cells. To gain insights into the location of these two proteins in living cells, we constructed an Tpo3- or Dur3-labeled strain with green fluorescent protein (GFP) at its C terminus under the control of its native promoter through homologous recombination (Supplementary Figures S3A,B). Using confocal fluorescence microscopy, we observed that the signals of Tpo3-GFP and Dur3-GFP were predominantly localized in the cell membrane. Occasionally, some weak Tpo3-GFP and Dur3-GFP signals were detected in the septa (Figure 5A). This subcellular location model suggested that Tpo3 and Dur3 mainly function in the cell membrane.

The overexpression of drug transporter genes in *A. fumigatus* is an important mechanism of azole resistance. To further analyze the importance of *tpo3* and *dur3* for the defense of *A. fumigatus* against ITC, we overexpressed the *tpo3* or *dur3* gene in the *A. fumigatus* wild-type strain A1160 using the *gpdA* (*Aspergillus nidulans* glyceraldehyde-3-phosphate dehydrogenase) promoter. The representative overexpression (OE) strains containing their native alleles were named OE*tpo3* and OE*dur3*, respectively, and are showed in Figure 5B. Quantitative reverse transcription-PCR (qRT-PCR) analysis determined that the mRNA levels of *tpo3* and *dur3* were sharply increased in these two OE strains (Figures 5C,D). Surprisingly, from the observed colony growth phenotypes on the plates, we found that both OE strains exhibited a slight defect in growth and presented heightened sensitivity to ITC (Figure 5B), suggesting that overexpression of *tpo3* or *dur3* was slightly toxic to *A. fumigatus* and caused an increase in the sensitivity to ITC.

Based on the above findings, we investigated whether the absence of *tpo3* or *dur3* could lead to increased tolerance to ITC. This analysis was carried out by gene deletion in the A1160 background strain, in which *tpo3* or *dur3* was replaced by the *Neurospora crassa pyr4* gene as a selection marker. Diagnostic PCR analyses showed the absence of the full-length sequences of *tpo3* and *dur3* in the deletion strains (Supplementary Figures S3C,D). The susceptibility levels of the *tpo3*- and *dur3*-null mutants and the wild-type parental strains to ITC were tested on plates. In contrast to the overexpression and the wild-type strains, the depletion strains (Figure 5B) both displayed increased growth rates in the presence of ITC. To confirm that the possible phenotypes result from the genes deletion rather than other unexpected mutations, the reconstituted strains were generated and named Δ*tpo3*:*tpo3*+ and Δ*dur3*:*dur3*+, respectively (Figure 5B). The reconstituted strains were confirmed by PCR (Supplementary Figure S3C) and the expression of *tpo3* or *dur3* was analyzed by qRT-PCR (Supplementary Figure S3E). Furthermore, the deletion of *dur3* in the Δ*tpo3* mutant created the double-deletion mutant (named Δ*tpo3*Δ*dur3*), which showed similar ITC tolerance to that of the Δ*tpo3* or Δ*dur3* single mutant, indicating a functional overlap of Δ*tpo3* and Δ*dur3* in regulating the response to ITC. Additionally, supplementation of polyamines can rescue the ITC effect between the deletion mutants and the wild-type strain (Figure 5B). Subsequently, we tested the MICs of deletion mutants, overexpression mutants, and the wild-type strain (A1160^C^) in response to ITC based on the CLSI-M38-A2 method. As shown in Supplementary Figure S4, the MICs of the deletion mutants and A1160^C^ strain were both 1 μg ml^–1^. Nevertheless, the deletion strains displayed accelerated hyphal growth compared to A1160^C^ strain in the presence of 0.5 μg ml^–1^ ITC, displaying tolerance toward ITC. In comparison, the MICs of the overexpression strains were 2-fold (0.5 μg ml^–1^ in the overexpression mutants versus 1 μg ml^–1^ in the wild-type strain) lower than that of the wild-type strain. This finding is consistent with the results of plate assay (Figure 5B). Taken together, these results demonstrated that *tpo3* and *dur3* are of important for susceptibility to ITC of *A. fumigatus*.

### Susceptibility to ITC of *A. fumigatus* Is Mediated by Intracellular Polyamines in a Dose-Dependent Manner

**FIGURE 6:**
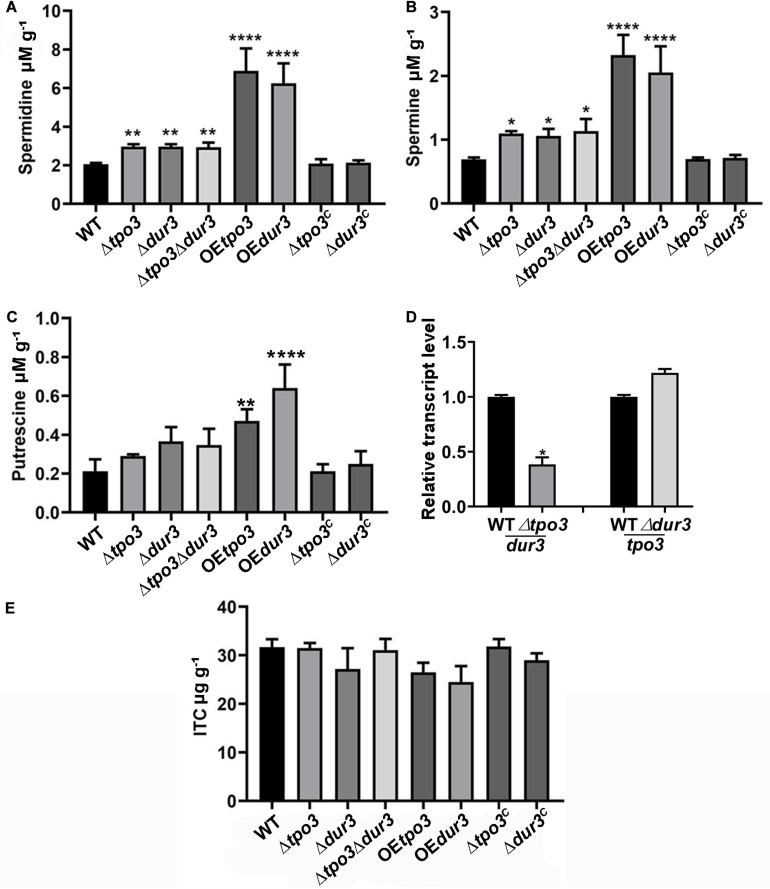
Deletion of *tpo3* and/or *dur3* accumulated moderate of polyamines while overexpression of *tpo3* or *dur3* accumulated overdose of polyamines in hyphal cells. Comparison of the intracellular concentrations of Spm **(A)**, Spd **(B),** and Put **(C)** in the displayed strains. **(D)** Gene expression level of *dur3* in Δ*tpo3* and gene expression level of *tpo3* in Δ*dur3*. **(E)** Intracellular ITC concentrations in strains stimulated by 1 μg ml^– 1^ ITC for 1 h. All the strains were cultivated in MMUU media. Polyamine and ITC concentration values were analyzed by one-way ANOVA with unpaired Student’s *t*-test and presented as the means ± SD of three biological samples. Error bars represent the corresponding standard deviations. *p < 0.05; **p < 0.01; ****p < 0.0001.

To identify the regulatory mechanism of the intracellular polyamine contents controlled by *tpo3* and *dur3*, we examined whether *tpo3* and *dur3* could regulate the intracellular levels of polyamines in *A. fumigatus*. We examined the contents of Put, Spd, and Spm in the parental wild-type, Δ*tpo3*, Δ*dur3*, and Δ*tpo3*Δ*dur3* strains, as well as in the overexpression strains, by HPLC. As shown in Figures 6A–C, the levels of endogenous Spd and Spm, but not Put, were moderately increased in all the tested *tpo3*- and/or *dur3*-deletion strains compared to the wild-type strain. We further investigated expression of *dur3* in the *tpo3* deletion mutant and vice versa. The *dur3* expression in the *tpo3* deletion mutant was decreased (Figure 6D). However, deletion of *dur3* had no influence on *tpo3* expression (Figure 6D). This result indicated that accumulation of intracellular polyamines in deletion strains was not caused by compensation expression between *tpo3* and *dur3*. In contrast, striking amounts of Spd, Spm, and Put were retained in the *tpo3-* or *dur3*-overexpressing strains. Moreover, we found that the accumulated polyamine with the highest level was Spd, suggesting the predominant role of Spd among these three endogenous polyamines.

A previous study demonstrated that *Catpo3* conferred resistance to azole drugs and polyamine homeostasis due to a direct effect of *Catpo3* in decreasing the intracellular accumulation of the antifungal clotrimazole (Costa et al., 2014). Here, we wondered whether Δ*tpo3* or Δ*dur3* can mediate drug efflux, one of the main mechanisms for drug resistance across fungal pathogens. Therefore, the deletion, OE, and wild-type strains were stimulated by ITC, and the intracellular accumulation of ITC in these strains was examined. With HPLC analysis, however, the results showed that no significant difference was found in the intracellular retention of ITC when comparing the deletion or OE mutants to the wild type strain, suggesting that the ITC tolerance of deletion mutants and the ITC sensitivity of the OE mutants did not result from differences in intracellular ITC transport by these strains (Figure 6E).

Taken together, these results indicated that *tpo3* or *dur3* makes a valuable contribution to the regulation of intracellular polyamine concentrations rather than the antifungal drug ITC. Moderately elevated intracellular polyamines could help to cope with ITC-induced stress, while high-dose polyamines combined with ITC inhibited the growth of *A. fumigatus*, indicating that polyamines function in a dose-dependent manner during ITC-induced stress, which was consistent with the plate test results above.

### Polyamines Are Involved in Susceptibility to ITC by Influencing ROS Generation

**FIGURE 7:**
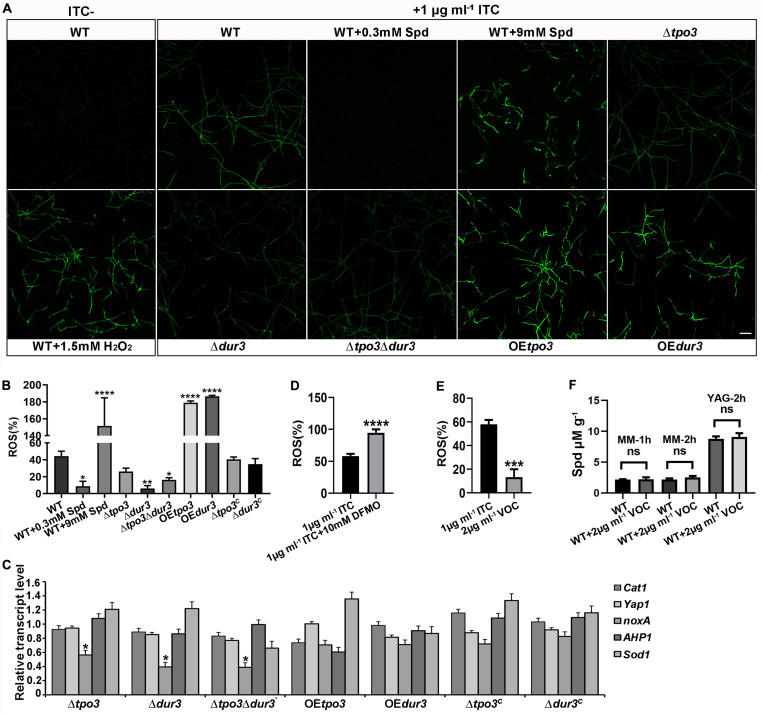
*tpo3* and *dur3* regulate ROS scavenging or generation. **(A)** ROS measurement in mycelia of the indicated strains. Moderate dose of polyamines can quench ITC-induced ROS while high dose of polyamines increased ITC-induced ROS accumulation. The untreated wild-type mycelia supplemented with or without 1.5 mM H_2_O_2_ were used as controls for comparison. All the mycelia were stained with DCFH-DA (2, 7-Dichlorofuorescin Diacetate). The images were obtained by confocal microscopy. Bars, 50 μm. The fluorescence intensity of DCFH-DA indicated the levels of total ITC-induced ROS in mycelia of indicated strains **(B)** and in mycelia of A1160^C^ in the presence or absence of 10 mM DFMO **(D)**. Fluorescence intensity values are presented as the means ± SD of three biological replicates and analyzed by one-way ANOVA with unpaired Student’s *t*-test (*p < 0.05; **p < 0.01; ****p < 0.0001). **(C)** Gene expression levels of genes (*Cat1*, *yap1*, *noxA*, *AHP1*, and *Sod1*) that encode for proteins involved in the scavenging or generation of ROS in different strains after 1 h of exposure to 1 μg ml^– 1^ ITC were measured by RT-qPCR. Beta-tubulin gene *tubA* was used as endogenous reference gene. Statistical analyses were performed by one-way ANOVA with unpaired Student’s *t*-test and the data were presented as the means ± SD of three independent experiments (*p < 0.05). **(E)** In contrast to ITC, VOC only induced limited amount of ROS in the wild-type A1160^C^. Statistical analyses were performed by one-way ANOVA with unpaired Student’s *t*-test and the data were presented as the means ± SD of three biological samples (***p < 0.001). **(F)** Intracellular Spd concentrations in WT (A1160^C^) and WT (A1160^C^) plus 2 μg ml^– 1^ VOC samples cultured in the indicated media and treated by VOC at different time points. Statistical analyses were performed by one-way ANOVA with unpaired Student’s *t*-test and the data were presented as the means ± SD of three biological samples.

Previous studies have indicated that antifungals induce ROS generation in fungal cells, which in general enhances the activity of antifungal agents (Kobayashi et al., 2002; Delattin et al., 2014; Liao et al., 2015; Shekhova et al., 2017). Polyamines are important for the defense against ROS, and they can act as ROS scavengers in plants and fungi (Chattopadhyay et al., 2006; Pottosin et al., 2014). To explain why intracellular polyamines in *A. fumigatus* play roles in susceptibility to ITC in a dose-dependent manner, we assessed ROS production in the hyphal cells by confocal fluorescence microscopy and measured the levels by a fluorescence microtiter plate reader after staining with the fluorescent dye DCFH-DA. As shown in Figures 7A,B, when *A. fumigatus* mycelia were incubated with 1 μg ml^–1^ ITC for 3 h, we found that the wild-type strain produced a high level of ROS. In contrast, 0.3 mM Spd-pretreated hyphal cells almost completely eliminated ROS. In addition, the ROS levels in these deletion strains were lower than those of the wild-type strain and were responsible for the ITC tolerance of the deletion strains (Figure 5B and Supplementary Figure S4). Moreover, our data showed that exposure to ITC resulted in extremely high levels of ROS in the hyphal cells of the OE*tpo3* and OE*dur3* strains, accounting for the enhanced sensitivity of these two overexpression mutants to ITC (Figure 4B). In comparison, there was no measurable difference in ROS content between the wild-type and OE strains under normal growth conditions (without ITC treatment) (Supplementary Figure S5), indicating that the high levels of ROS in the OE strains after treatment of ITC were mainly caused by ITC rather than over-dose of polyamine.

Since *Cat1* (hyphal catalase, AFUB_046060), *yap1* (AFUB_075990), *noxA* (superoxide-generating NADPH oxidase, AFUB_001420), *AHP1*/*aspf3* (thioredoxin peroxidase, AFUB_096050), and *Sod1* (superoxide dismutase, AFUB_056780) have been found to be closely related to ROS production or elimination (Lara-Ortiz et al., 2003; Paris et al., 2003; Lessing et al., 2007; Lambou et al., 2010), we investigated the transcript levels of these orthologous genes by qRT-PCR analysis in the wild-type, deletion, and OE strains after exposure to 1 μg ml^–1^ ITC. As shown in Figure 7C, all three deletion strains exhibited a significant downregulation of *noxA* gene expression, which has been shown to produce ROS (Lara-Ortiz et al., 2003). The significantly decreased transcription level of *noxA* in all deletion strains can be at least partly responsible for the reduction in ROS levels.

Furthermore, investigation of the ROS content in *A. fumigatus* A1160^C^ under combination of DFMO and ITC showed excessive accumulated ROS in hyphal cells (Figure 7D), suggesting that inhibition of polyamine biosynthesis reduced the level of intracellular polyamines (Figure 3B), inhibited the elimination of ROS induced by ITC, and finally enhanced the activity of ITC against *A. fumigatus* through ROS-induced oxidative injury.

To date, the ability of VOC to produce ROS in fungal cells has yet to be studied. In addition, we wondered why the antifungal efficacy of ITC rather than VOC can be decreased by polyamines. To this end, we examined ROS generation when VOC was used to treat *A. fumigatus* hyphal cells. As shown in Figures 7E,F, VOC treatment induced lower levels of ROS and could not change the level of Spd than ITC treatment, indicating that the mechanism of killing was mainly dependent on the specific mode of its action rather than ROS induction.

## Discussion

As one of the most common pathogenic species, *A. fumigatus* is generally susceptible to azoles. However, intrinsic and acquired resistance in this human opportunistic fungus is well documented, raising concerns in recent years (Snelders et al., 2008; Arendrup, 2014; Zavrel and White, 2015; Rybak et al., 2019). Cellular metabolism and metabolic regulation are importance for biofilm formation (Fanning and Mitchell, 2012; Liao et al., 2015; Long et al., 2018), which is a common drug-resistance factor. By applying a metabolomics approach, limited researches with the aim of exploring new mechanisms underlying the resistance to azoles have confirmed that amino acid metabolism was high in *C. albicans* or *A. fumigatus*. In addition, lipids and carbohydrates were significantly changed (Li et al., 2018; Long et al., 2018). In this study, we identified some potential small molecular metabolites that might function in the response to ITC. Consistent with these previous findings, the amino acid contents were significantly altered in this study when mycelial cells were treated with ITC (Figure 1 and Supplementary Table S3).

Spd, one metabolite identified in the ITC treatment strains, had a 1.35-fold higher content than the controls. However, two other polyamines, Put and Spm, were not detected. This finding result from the fact that Put and Spm act at low concentrations and Spd is usually the major polyamine in fungal cells (Walters et al., 1997). Indeed, the HPLC analysis shown in Figures 6A–C proved that Spd was more abundant than Spm and Put. Notably, arginine, functioning as a starting material for the biosynthesis of polyamines, also had an approximately 1.97-fold increase in content. However, findings from *C. albicans* have shown that Spm was reduced in the presence of fluconazole (Li et al., 2018). This result may be attributed to the different fungal species, the different drugs or the different drug stimulus conditions. In addition, our results showed that the addition of Spd, Put, and Spm significantly influenced the resistance of *A. fumigatus* to ITC (Figure 2A,C). Taken together with our results, these data indicated that cellular polyamines were associated with drug resistance. Furthermore, some interesting metabolites, such as lysoPCs, were reduced in the ITC-treated strains. Consistent with our results, resistant strains of *C. albicans* showed decreased contents of LysoPC, LysoPE, and LysoPG (Li et al., 2018). LysoPCs were reported to evoke cell membrane permeabilization (Colles and Chisolm, 2000). Therefore, these results implied that during contact with ITC, fungal cells can degrade LysoPCs to alleviate a possible synergetic effect on the cell membrane.

Coupled with our comparative RNA-seq analysis of *A. fumigatus* under the two conditions applied above (ITC treatment and non-treatment), we found that ITC treatment of *A. fumigatus* altered the expression of thousands of genes (Figure 4A and Supplementary Table S4), showing the magnitude of gene modulation in *A. fumigatus* during adaptation to ITC-induced stress. We analyzed the RNA-seq data and identified two genes upregulated at the transcriptional level, *tpo3* (had a 3.03-fold increase) and *dur3* (had a 2.15-fold increase), which are two orthologs of the yeast polyamine transporters *tpo3* and *dur3* and are predicted to be involved in polyamine transport. For many opportunistic pathogens, the balance (synthesis and transport) of intracellular polyamines is vital for growth in adverse environmental and drug stress conditions (Tati et al., 2013; Bleackley et al., 2014; Costa et al., 2014; Pottosin et al., 2014; Liao et al., 2015; Rocha and Wilson, 2019). ABC polyamine transporters, important regulators of intracellular polyamine homeostasis, play important roles in drug resistance (Tati et al., 2013; Bleackley et al., 2014; Costa et al., 2014).

We wondered whether *tpo3* and *dur3* were specific polyamine transporters or ITC drug transporters. Our data indicated that *tpo3* and *dur3* regulate cellular polyamine homeostasis rather than ITC transport. This finding is supported by several lines of evidence: first, BLASTP and TMHMM software analysis showed that Tpo3 and Dur3 were both conserved transmembrane proteins related to polyamine homeostasis (Supplementary Figure S2). GFP-tagging experiments showed that both *tpo3* and *dur3* were localized in the plasma cell membrane (Figure 5A). Second, depletion of *tpo3* and/or *dur3* caused moderate accumulation of polyamines, suggesting that pathways related to polyamine synthesis or transport in *A. fumigatus* could cooperatively and compensatorily regulate the polyamine contents in the absence of *tpo3* and/or *dur3*. In contrast, constitutive overexpression of *tpo3* or *dur3* by the *gpdA* promoter disturbed polyamine homeostasis, accumulated high levels of polyamines and was cytotoxic to cells (Figures 5B, 6A, 7A; Rocha and Wilson, 2019). Third, intracellular ITC detection experiments showed that *tpo3* and *dur3* are dispensable for ITC extrusion (Figure 6E), although the transport of clotrimazole by *Catpo3* was observed in *C. albicans* in previous research (Costa et al., 2014). In conclusion, these results strongly demonstrated that *tpo3* and *dur3* specifically regulate polyamine homeostasis in *A. fumigatus*.

ROS are a variety of molecules derived from molecular oxygen that can cause oxidative damage to DNA, proteins and lipids and eventually cause cell death (Cap et al., 2012). Apart from the specific mode of action on ergosterol, azoles generally have ROS-inducing effects in susceptible fungi (Delattin et al., 2014). In this regard, reports have shown that the induced ROS contributed to the antimycotic effects of antifungal agents (Delattin et al., 2014). By contrast, quenching or reducing azole-induced ROS accumulation resulted in a significant decrease in azole antifungal activity and contributed to drug resistance (Delattin et al., 2014; Li et al., 2019). Likewise, the dysregulation of intracellular polyamine content in *A. fumigatus* is correlated with the ROS level and eventually influenced susceptibility to ITC in our study. Appropriate amount of polyamines can act as ROS scavenger, and this is why addition of polyamines was able to increase ITC resistance significantly (Figures 2A, 7A,B). Excessive amount of polyamines alone was cytotoxic, and this cytotoxic effect may be caused by factors such as an increase of plasma membrane polarization or binding anionic macromolecules (Costa et al., 2014; Liu et al., 2015). As a result, a synergistic effect was achieved predominantly when ITC was combined with excessive amount of polyamines, regardless of whether excessive amount of polyamines can eliminate ROS. Ultimately, overdose of polyamines can enhance the effect of ITC on antifungal activity and ROS production (Figures 7A,B). Moreover, VOC treatment induced lower levels of ROS than ITC treatment, confirming that polyamines can specifically protect cells from ITC via elimination of ITC-induced ROS accumulation. Most importantly, through plate tests, HPLC analysis of polyamines and ROS assays, we established a link among polyamine homeostasis, ROS level, and susceptibility to ITC: inhibition of polyamine biosynthesis caused reduction of polyamines, production of excessive amounts of ROS during ITC-induced stress, and enhanced ITC sensitivity; ITC treatment resulted in the upregulation of the expression of *tpo3* and *tpo3*, mild accumulation of Spd in wild-type cells, and production of a certain amount of ROS; deletion of *tpo3* and/or *tpo3* caused moderate accumulation of Spm and Spd, production of a limited amount of ROS during ITC-induced stress, and ITC tolerance; and overexpression of *tpo3* or *dur3* resulted in excessive accumulation of polyamines, excessive levels of ROS during ITC-induced stress, and enhanced sensitivity to ITC.

## Conclusion

This study provides the first characterization of polyamines and their putative cell-membrane regulators *tpo3* and *dur3* in the filamentous fungus *A. fumigatus* and elucidation of their important roles in regulating ROS levels and susceptibility to ITC. Inhibition of polyamine biosynthesis caused ROS accumulation and enhanced ITC sensitivity; a mild increase in cellular polyamines during ITC-induced stress can help *A. fumigatus* adapt to this stress; depletion of *tpo3* and/or *dur3* resulted in moderate accumulation of polyamines, which conferred tolerance to ITC through ROS scavenging; and overexpression of *tpo3* and *dur3* greatly increased the intracellular polyamine content and enhanced the antifungal activity of ITC through overgeneration of ROS. Our study revealed an underlying mechanism of action of *tpo3* and *dur3* on susceptibility to ITC in a concentration-dependent manner: expression of *tpo3* and *dur3* → polyamine homeostasis → ROS level → ITC susceptibility. This study will improve novel insights into the function of polyamines and enhance our ability to control the activities and drug resistance of fungal pathogens.

## Data Availability Statement

The datasets presented in this study can be found in online repositories. The names of the repository/repositories and accession number(s) can be found below: EMBL-EBI MetaboLights database, accession no: MTBLS1739.

## Author Contributions

LL and GZ designed the work. MC, GZ, and SW completed the experiments. LL, GZ, MC, JZ, and LT evaluated and analyzed the results. GZ and MC wrote the manuscript. All authors approved the final manuscript.

## Conflict of Interest

The authors declare that the research was conducted in the absence of any commercial or financial relationships that could be construed as a potential conflict of interest.

## Footnotes

1 https://www.ebi.ac.uk/metabolights/

2 http://www.ncbi.nlm.nih.gov/sra

3 http://www.genome.jp/kegg/

4 http://www.cbs.dtu.dk/services/TMHMM/
