# Supplementary material for: tpo3 and dur3, Aspergillus fumigatus Plasma Membrane Regulators of Polyamines, Regulate Polyamine Homeostasis and Susceptibility to Itraconazole
Source: Front Microbiol. 2020 Dec 16;11:563139. doi: 10.3389/fmicb.2020.563139 (PMC7772357; doi:10.3389/fmicb.2020.563139)
Supplement: Supplementary file 1 [file Table_1.DOCX]

**Supplementary Table S1 |** *Aspergillus fumigatus* strains used in this study.

| **Strain** | **Genotype** | **Reference** |
| --- | --- | --- |
| A1160 | Δ*KU80*, *pyrG1* | FGSC |
| A1160^C^ | Δ*KU80*, *A1160::pyrG1* | This study |
| Δ*tpo3* | Δ*KU80*, *pyrG1*, Δ*tpo3::pyr4* | This study |
| Δ*dur3* | Δ*KU80*, *pyrG1*, Δ*dur3::pyr4* | This study |
| Δ*tpo3::tpo3+* | Δ*KU80*, *pyrG1*, Δ*tpo3::tpo3* | This study |
| Δ*dur3::dur3+* | Δ*KU80*, *pyrG1*, Δ*dur3::dur3* | This study |
| Δ*tpo3*Δ*dur3* | Δ*KU80*, *pyrG1*, Δ*tpo3::pyr4,* Δ*dur3::hph* | This study |
| OE*tpo3* | Δ*KU80*, *pyrG1*, *gpdA(p)-tpo3, hph* | This study |
| OE*dur3* | Δ*KU80*, *pyrG1*, *gpdA(p)-dur3*, *hph* | This study |
| Tpo3-GFP | *ΔKU80, pyrG1, tpo3::gfp::pyrG1* | This study |
| Dur3-GFP | *ΔKU80, pyrG1, dur3::gfp::pyrG1* | This study |
