# Supplementary material for: tpo3 and dur3, Aspergillus fumigatus Plasma Membrane Regulators of Polyamines, Regulate Polyamine Homeostasis and Susceptibility to Itraconazole
Source: Front Microbiol. 2020 Dec 16;11:563139. doi: 10.3389/fmicb.2020.563139 (PMC7772357; doi:10.3389/fmicb.2020.563139)
Supplement: Supplementary file 2 [file Table_2.DOCX]

**Supplementary Table S2 |** All primers used in this study.

| **Primer name** | **Primer sequence 5’-3’** |
| --- | --- |
| tpo3 P1 | GGCTCACAGTCGCCATCC |
| tpo3 P2 | CATGTGGGAATGGCCGCC |
| tpo3 P3 | GGCTTGTCTGCTCCCGGGTGGGAGGTAGCCTGAG |
| tpo3 P4 | ACGCCAGGGTTTTCCCCAATAGCAGCATGCAATATAGCG |
| tpo3 P5 | GTGTCTTGGGCGAGTCGAG |
| tpo3 P6 | ACAAGCCCAGCCGGTAAC |
| Diagtpo3 | GATGGTGTACAGTCCGAGCG |
| Diagdur3 | CATTCGTCGCGTAGGCGG |
| dur3 P1 | GGCTCTCGTTACATGCTACTCG |
| dur3 P2 | CTATTGGCATCGGCCAGCT |
| dur3 P3 | GGCTTGTCTGCTCCCGGTTTGACAGCCTCTAGTTCCC |
| dur3 P4 | ACGCCAGGGTTTTCCCGCCACTGTGTTGGGATCC |
| dur3 P5 | GCCAACACTGGAACGGACAC |
| dur3 P6 | CCTCGATGCTGGAAGGGTAC |
| tpo3-com S1 | GGCTCACAGTCGCCATCC |
| tpo3-com S2 | ACAAGCCCAGCCGGTAAC |
| dur3-com S1 | GGCTCTCGTTACATGCTACTCG |
| dur3-com S2 | CCTCGATGCTGGAAGGGTAC |
| DiagcDNAtpo3 S | GACGCTCCATGAAGCTGG |
| DiagcDNAtpo3 A | GCCGATCAGAATCGGGATG |
| DiagcDNAdur3 S | CAGCCTGTTTCCTACTGCCC |
| DiagcDNAdur3 A | GACCAACGACCACCCAGC |
| dur3 hph P1 | GGCTCTCGTTACATGCTACTCG |
| dur3 hph P2 | ACTGGAGCATCACTCAATCA |
| dur3 hph P3 | CCTGTGTGTAGAGATACAAGGGAATTCGTTTGACAGCCTCTAGTTCCC |
| dur3 hph P4 | CACTCCACATCTCCACTCGAGCCACTGTGTTGGGATCC |
| dur3 hph P5 | CCAACACTGGAACGGACA |
| dur3 hph P6 | CCTCGATGCTGGAAGGGTAC |
| tpo3-gfp P1 | CATTGCGTTCTTCGCCGC |
| tpo3-gfp P2 | CGGCGTGGTCATGATCGT |
| tpo3-gfp P3 | CCAGCGCCTGCACCAGCTCCATGCTTTTCAAATTTCGCCCG |
| tpo3-gfp P4 | CATCAGTGCCTCCTCTCAGACAGCAATAGCAGCATGCAATATAGCG |
| tpo3-gfp P5 | GTGTCTTGGGCGAGTCGAG |
| tpo3-gfp P6 | ACAAGCCCAGCCGGTAAC |
| dur3-gfp P1 | GGCAGTTACTTCGGCCTTCTC |
| dur3-gfp P2 | CTCTACTACGCCGGAATTGGT |
| dur3-gfp P3 | CCAGCGCCTGCACCAGCTCCAATGCTTGTCTTTGGCTCCATC |
| dur3-gfp P4 | CATCAGTGCCTCCTCTCAGACAGAGTTCTTCTCCGTGGAAGGAAT |
| dur3-gfp P5 | GCCAACACTGGAACGGACAC |
| dur3-gfp P6 | CCTCGATGCTGGAAGGGTAC |
| Diagtpo3gfp S | CATTGCGTTCTTCGCCGC |
| Diagdur3gfp S | GGCAGTTACTTCGGCCTTCTC |
| Diaggfp A | TAGGGACCGAGACCTGTATC |
| gfp+pyrg F | GGAGCTGGTGCAGGCGCTGG |
| gfp+pyrg R | CTGTCTGAGAGGAGGCACTGATG |
| pyr4 F | TGGCGTTACCCAACTTAATCG |
| pyr4 R | GCTTTCGGGAACTGGCTACTTAT |
| hph F | GAATTCCCTTGTATCTCTACACACAGGC |
| hph R | TCGAGTGGAGATGTGGAGTGGGCGCTTA |
| OE-tpo3 F | ACCTTTAATCAAGCTTATCGATATGTCGGACCTACCTCCG |
| OE-tpo3 R | CCTCGAGGTCGACGGTATCGATTTAATGCTTTTCAAATTTCGCCCG |
| OE-dur3 F | ACCTTTAATCAAGCTTATCGATATGTCCTCAGAGGAGGTCAAG |
| OE-dur3 R | CCTCGAGGTCGACGGTATCGATTCAAATGCTTGTCTTTGGCTCC |
| RT-tubA F | ACGTTACCTCACCTGCTCTGC |
| RT-tubA R | GATGTTGTTGGGAATCCACTCA |
| RT-tpo3 F | GCTGGTACGTCACCATTGTC |
| RT-tpo3 R | ACAGAGGACTCCAGAGCAAC |
| RT-dur3 F | CATTACCTGGGCTTTGAGGAG |
| RT-dur3 R | AACCCGACTTTACAGACCGT |
| RT- AHP1 F | CAACTACAACGCCTCCAAGG |
| RT- AHP1 R | TCTCGATGTACTCGGGAACG |
| RT-NoxA F | CTTTGTGCGAGACACTCCAG |
| RT-NoxA R | TACCAGTTCCCACCTCCAAC |
| RT-Cat1 F | CCGATTCGTGACAGATGACG |
| RT-Cat1 R | ATTCTTGCCAGAGGTCTGCT |
| RT-Sod1 F | ACCACCGTCTCTTGGAACAT |
| RT-Sod1 R | TTACCCTCAGCATCGGTCTC |
| RT-Yap F | CATCTACACGACCTGGCTCT |
| RT-Yap R | GGTGAGGTCCCATTGGAAGA |
