# Supplementary material for: tpo3 and dur3, Aspergillus fumigatus Plasma Membrane Regulators of Polyamines, Regulate Polyamine Homeostasis and Susceptibility to Itraconazole
Source: Front Microbiol. 2020 Dec 16;11:563139. doi: 10.3389/fmicb.2020.563139 (PMC7772357; doi:10.3389/fmicb.2020.563139)
Supplement: Supplementary file 5 [file Table_5.DOCX]

| **Transcript ID** | **Gene product** | **Fold change**  **(WT-1ITC/WT)** |
| --- | --- | --- |
| **Putative** **polyamines biosynthesis genes** | | |
| AFUB_052180 | Spermidine biosynthetic process, spermine biosynthetic process | 1.00 |
| AFUB_065110 | Putrescine biosynthetic process, spermidine biosynthetic process | 0.57 |
| AFUB_012980 | Spermidine synthase activity | 1.00 |
| **Putative polyamines transporters** | | |
| AFUB_005210 | Role in putrescine, spermidine and urea transport | **2.15** |
| AFUB_101650 | Spermine transport, major facilitator superfamily multidrug transporter | **3.03** |
| AFUB_099680 | Putrescine transmembrane transporter activity, putrescine transport | 1.47 |
| AFUB_089830 | Polyamine transport, putative amino acid permease | 0.55 |
| AFUB_070570 | Polyamine transmembrane transporter activity | 0.93 |
| AFUB_011780 | Polyamine transport, putative GABA permease | 1.09 |
| **Putative polyamine oxidases** | | |
| AFUB_094780 | Flavin containing polyamine oxidase, oxidoreductase activity | 1.27 |
| AFUB_057270 | Flavin containing polyamine oxidase, oxidoreductase activity | 0.93 |

**Supplementary Table S5** **|** The annotated genes corresponding to the expression of genes encoding proteins putatively involved in polyamine transporters and biosynthesis related genes.

*Genes upregulated > 2-fold are in bold.*
