# Supplementary material for: tpo3 and dur3, Aspergillus fumigatus Plasma Membrane Regulators of Polyamines, Regulate Polyamine Homeostasis and Susceptibility to Itraconazole
Source: Front Microbiol. 2020 Dec 16;11:563139. doi: 10.3389/fmicb.2020.563139 (PMC7772357; doi:10.3389/fmicb.2020.563139)
Supplement: Supplementary file 6 [file Data_Sheet_1.DOCX]

Supplementary Material

## Supplementary Figures


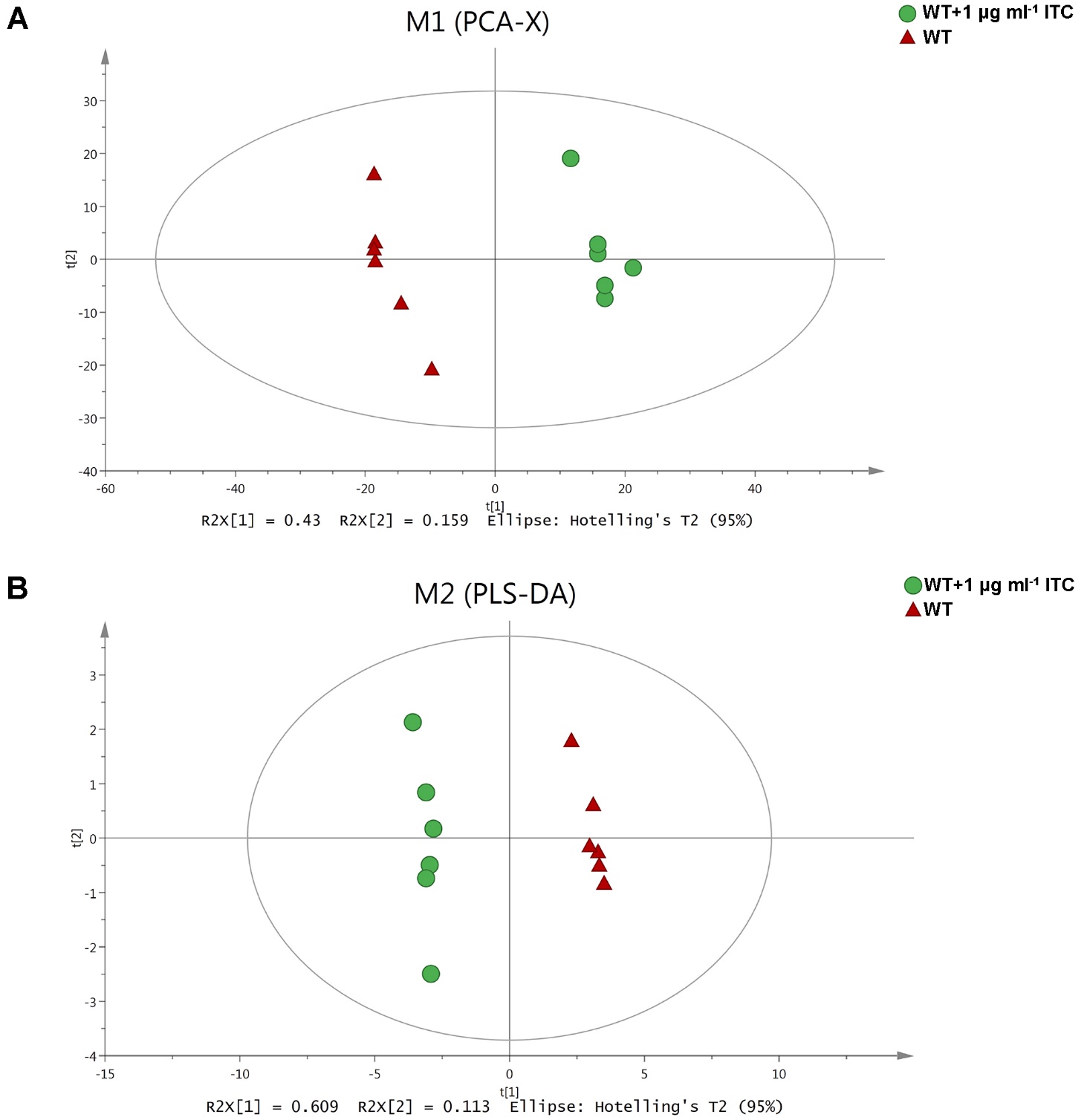


**Supplementary Figure S1 |** PCA **(A)** and PLS-DA **(B)** score plots of intracellular metabolites between the WT+1 μg ml^-1^ ITC group vs WT group from LC-MS.


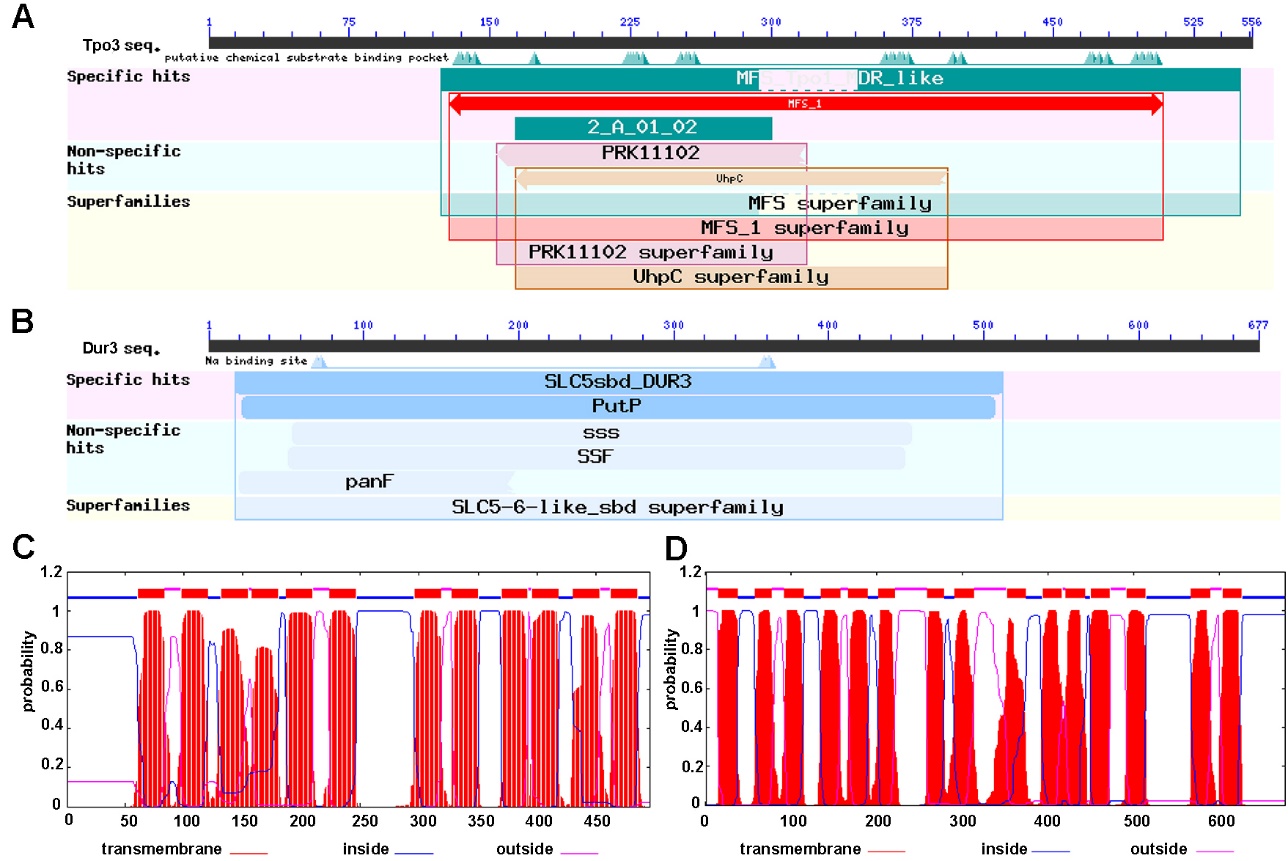


**Supplementary Figure S2 |** Schematic diagram of conserved domains of Tpo3 **(A)** and dur3 **(B)** in *A. fumigatus* based on the BLASTP tool. Tpo3 **(C)** and Dur3 **(D)** are predicted integral membrane protein with 12 and 15 transmembrane spans, respectively. The probability of the transporters exhibiting transmembrane α-helical segments, based on its hydrophobicity, was calculated using the TMHMM online tool.


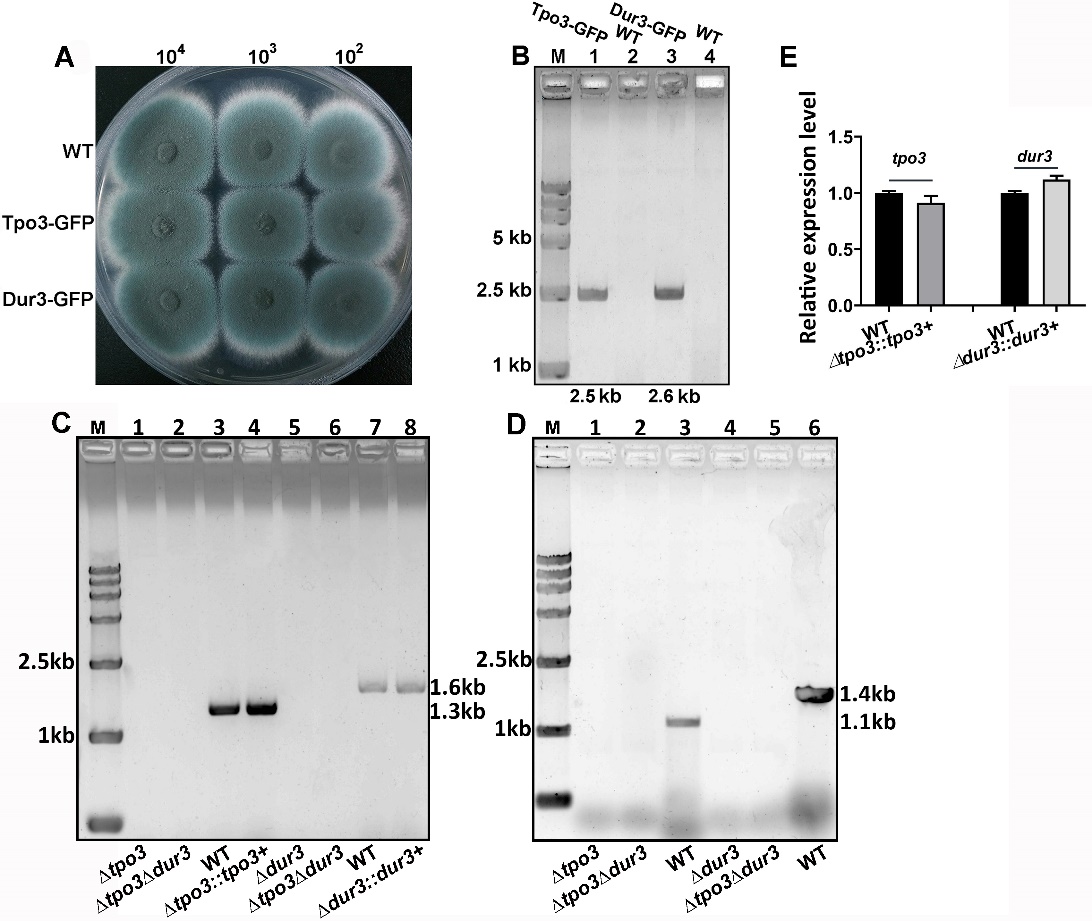


**Supplementary Figure S3 |** Construction of GFP-labeled strains and deletion strains. **(A)** Phenotypic characterization of the Tpo3-GFP and Dur3-GFP strains. **(B)** Diagnostic PCR confirmed that the full-length sequence of *tpo3* or *dur3* was replaced by fusion cassette in the indicated strain. For lanes 1 and 2, PCR primer pair was Diagtpo3gfp S/Diaggfp A, to detect whether there was *tpo3*-*gfp* fragment, and the expected size was 2.5 kb; for lanes 3 and 4, the PCR primer pair was Diagdur3gfp S/Diaggfp A, to detect whether *dur3*-*gfp* fragment exists in the genome, and the expected size was 2.6 kb. **(C)** Diagnostic PCR using genomes as templates confirmed that the full-length sequence of *tpo3* or *dur3* was replaced by selection gene in the deletion strains and was complemented in the reconstitution strains. For lanes 1, 2, 3 and 4, PCR primer pair was tpo3 P2/Diagtpo3, to detect whether there was *tpo3* gene, and the expected size was 1.3 kb; for lanes 5, 6, 7 and 8, the PCR primer pair was dur3 P2/Diagdur3, to detect whether *dur3* still exists in the genome, and the expected size was 1.6 kb. **(D)** PCR analysis using cDNAs as templates showed that the full-length sequence of *tpo3* or *dur3* was deleted in the indicated mutants. For lanes 1, 2 and 3, the PCR primer pair was DiagcDNAtpo3 S/DiagcDNAtpo3 A, to detect whether *tpo3* still existed in the cDNAs, and the expected size was 1.1 kb. For lanes 4, 5 and 6, the PCR primer pair was DiagcDNAdur3 S/DiagcDNAdur3 A, to detect whether *dur3* still existed in the cDNAs, and the expected size was 1.4 kb. **(E)** Real-time PCR analysis demonstrated the normal expression level of *tpo3* or *dur3* in the reconstitution strains.


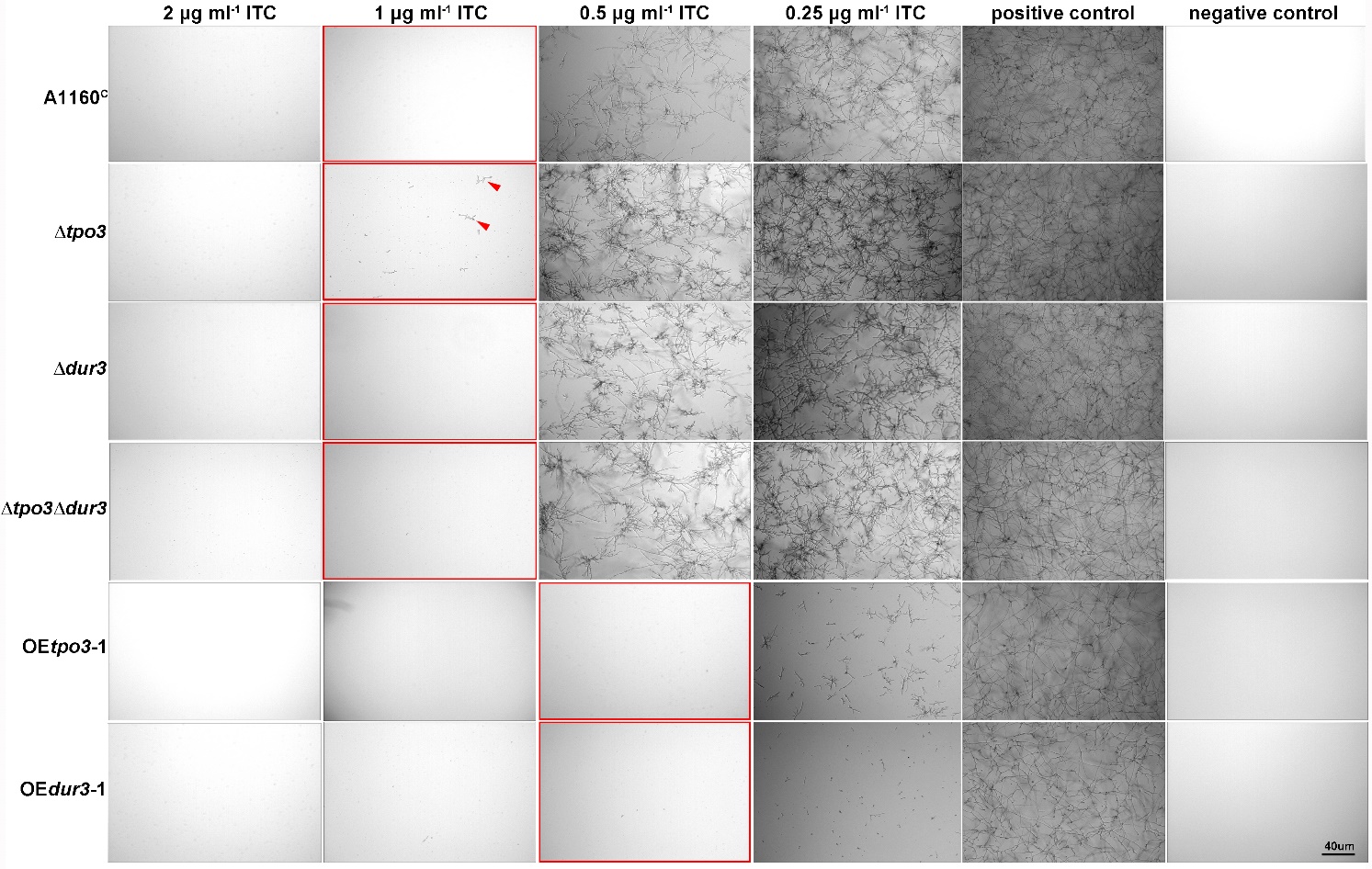


**Supplementary Figure S4 |** Microdilution assay for indicated strains. Growth is shown after 48 h at 35℃ in RPMI 1640 media plus 5 mM uridine and 10 mM uracil. Minimal inhibitory concentration (MIC) values of ITC for the respective strains were determined by microscopy and marked in red boxes. Arrowheads indicated some conidial germlings of *A. fumigatus.* Bars, 40 μm.


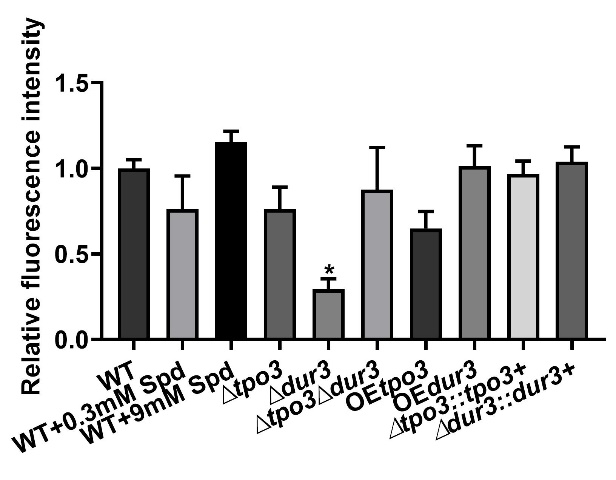


**Supplementary Figure S5 |** Fluorescence intensity of DCFH-DA indicated the levels of total ROS in mycelia of indicated strains without ITC treatment. Fluorescence intensity values are presented as the means ± SD of three biological replicates and analyzed by one-way ANOVA with unpaired Student’s t-test (*p < 0.05).
